# A Bayesian model for classifying all differentially expressed proteins simultaneously in 2D PAGE gels

**DOI:** 10.1186/1471-2105-13-137

**Published:** 2012-06-19

**Authors:** Steven H Wu, Michael A Black, Robyn A North, Allen G Rodrigo

**Affiliations:** 1 Bioinformatics Institute, University of Auckland, Private Bag, 92019, Auckland, New Zealand; 2 School of Biological Sciences, University of Auckland, Private Bag, 92019, Auckland, New Zealand; 3 Department of Biochemistry, University of Otago, P. O. Box 56, Dunedin, New Zealand; 4 Women's Health Academic Centre, King’s College London, London, UK; 5 Biology Department, Duke University, Duke Box, 90338, Durham, NC, 27708, USA; 6 The National Evolutionary Synthesis Center, Durham, NC, 27705, USA

**Keywords:** Two-dimensional polyacrylamide gel electrophoresis (2D PAGE), Global Bayesian model, Differentially expressed protein, Markov chain Monte Carlo (MCMC)

## Abstract

**Background:**

Two-dimensional polyacrylamide gel electrophoresis (2D PAGE) is commonly used to identify differentially expressed proteins under two or more experimental or observational conditions. Wu et al (2009) developed a univariate probabilistic model which was used to identify differential expression between Case and Control groups, by applying a Likelihood Ratio Test (LRT) to each protein on a 2D PAGE. In contrast to commonly used statistical approaches, this model takes into account the two possible causes of missing values in 2D PAGE: either (1) the non-expression of a protein; or (2) a level of expression that falls below the limit of detection.

**Results:**

We develop a global Bayesian model which extends the previously described model. Unlike the univariate approach, the model reported here is able treat all differentially expressed proteins simultaneously. Whereas each protein is modelled by the univariate likelihood function previously described, several global distributions are used to model the underlying relationship between the parameters associated with individual proteins. These global distributions are able to combine information from each protein to give more accurate estimates of the true parameters. In our implementation of the procedure, all parameters are recovered by Markov chain Monte Carlo (MCMC) integration. The 95% highest posterior density (HPD) intervals for the marginal posterior distributions are used to determine whether differences in protein expression are due to differences in mean expression intensities, and/or differences in the probabilities of expression.

**Conclusions:**

Simulation analyses showed that the global model is able to accurately recover the underlying global distributions, and identify more differentially expressed proteins than the simple application of a LRT. Additionally, simulations also indicate that the probability of incorrectly identifying a protein as differentially expressed (i.e., the False Discovery Rate) is very low. The source code is available at https://github.com/stevenhwu/BIDE-2D.

## Background

Two-dimensional polyacrylamide gel electrophoresis (2D PAGE) separates hundreds or thousands of proteins simultaneously by their isoelectric point and molecular weight [1]. There are two main approaches to analyse 2D PAGE: (1) an image-based approach, which analyses the raw or preprocessed gel images [2,3], and (2) a spot-based approach, whereby a standard analytical pipeline is used to identify up- or down-regulated proteins by gel scanning, spot-detection and spot-matching using appropriate software [4,5]. Data obtained are expressed as absolute or relative protein intensities, typically transformed into log-values. By detecting statistically significant differences in the spot intensities under different experimental or sampling conditions, 2D PAGE is a useful technique for exploring potentially differentially expressed proteins.

Most of the commercial packages for 2D PAGE analysis include several standard statistical analysis methods, for example, two-sample Student's *t*-tests, Analysis of Variance, and Principal Component Analysis [6,7]. Nonetheless, a significant challenge with most 2D PAGE analyses is the problem of missing values, whereby spots on one gel are not identified, or matched with, spots on another gel [8]. This should not come as a surprise: the expression of proteins varies from individual to individual from one experimental condition to the next, along with technical variation between gels. Previously, we proposed a likelihood-based model that identified differentially expressed proteins, and which accounted for missing values by positing a class of proteins where the probability of non-expression is greater than zero [9]. In particular, we divided missing values into two categories, due either to the non-expression of a protein, or a level of expression that fell below the limit of detection [3,10]. The likelihood function utilized a mixture of the two probabilistic models, thus allowing both possible causes of missing values. By applying a Likelihood Ratio Test (LRT), we classified a protein as “differentially expressed” if there was statistically significant support for either a difference in mean expression intensities or a difference in the probabilities of expression across the two categories.

In this paper, we extend our univariate likelihood model to a global model. The aim of a global model is to utilize the relationship between spots so that information about expression probabilities and differences in mean expression intensities can be modeled coherently across all spots. The global likelihood model proposed in this paper maintains all the advantages of the local model proposed previously, that is, the incorporation in the model of probabilities of expression and a limit of detection. Additionally, the global model includes several parametric probability functions that deliver the expected probability of expression and mean expression intensities for individual spots. In other words, the probability of expression and the mean expression intensity for any given spots are random variables drawn from global distributions of these variables, and the parameters of these global distributions are estimated from all expression data. While the characterization and use of global distributions of expression frequencies and intensities is not novel [11,12], this is the first time that this type of approach has been applied to the problem of modeling protein abundance in 2D PAGE. The empirical distributions of these data sets lend themselves to approximations by well-studied statistical distributions, and their use in statistical inference delivers greater power to detect differentially expressed spots. We illustrate the properties of the global model using simulated data, where the true parameters of the probabilities of expression, and the mean expression intensities are known.

## Methods

### The Global Bayesian Model

In our paper, the global model is applied to a case–control experimental design, where subjects belong to either a Case (disease) or Control group. Under the simplest experimental design, individuals are assigned to either the Case or Control group, and each subject has a sample that is processed using 2D-PAGE. This approach produces as many 2D-PAGE gels as there are subjects, and after application of the appropriate software algorithms, a list of “spots” is produced (corresponding to proteins that were expressed on at least one gel), along with the intensities of these spots for each gel. Before any analysis is carried out, we calculate the relative intensities by dividing the intensity of individual spots by the sum of all intensities on the corresponding gel, followed by *log*_*2*_ transformation. In many instances, there will be no intensity value for a given protein, indicating (as previously noted), that the spot was not expressed or not detected. These spots are indicated by “NA” in the dataset.

The global model proposed here is a hierarchical model with two layers. The first layer is referred to as the local layer. This layer calculates the likelihood for an individual protein, with each protein having its own parameters. The second or “global” layer connects all parameters from the local layers together. Parameters associated with this layer are referred to as global parameters. Since the model attempts to recover a large number of parameters, it is analytically and computationally cumbersome to obtain estimates within a likelihood-based framework. Instead, we have chosen to use Bayesian Markov chain Monte Carlo (MCMC) integration (described below), which is a computationally tractable approach. More importantly, Bayesian MCMC integration allows us to specify prior probability distributions that capture what we expect our parameters to look like when there is no difference between Case and Control. Since the point of Bayesian inference is to recover the posterior distribution (i.e., the distribution of the model parameters, after the incorporation of new data), any significant deviation between the posterior and the prior distributions is a signal that there are statistical differences between Cases and Controls.

### The local layer

The local layer focuses on the expression of an individual spot and can be described by four parameters. These four parameters are: 1) the mean for control group expression intensity *μ*, 2) the difference between case and control mean expression intensities *δ*, (i.e., the mean for the case group is calculated by *μ*_*1*_ *= μ*_*0*_ *+ δ)*, 3) the probability of expression for the control group *p*_*0*_, which can be expressed as a function of *κ* and 4) the difference between probabilities of expression between the two groups, *τ*. The probabilities of expression for the Control and Case groups are calculated by $p_{0}=\frac{\exp\left(\kappa \right)}{1+\exp\left(\kappa \right)}$ and $p_{1}=\frac{\exp\left(\kappa +\tau \right)}{1+\exp\left(\kappa +\tau \right)}$ respectively. Both groups are assumed to have the same standard deviation for expression intensities, σ_s_, the details of which will be discussed later.

The likelihood of a parameter is defined as the probability of obtaining the observed data given a specified value of that parameter. Let *L(Θ*_*s*_*)* be the likelihood associated with the expression intensity of protein *s* on the gel, where *Θ*_*s*_ *= (μ*_*s*_*, δ*_*s*_*, κ*_*s*_*, τ*_*s*_*, σ*_*s*_*, d)*, and the subscripts denote parameters specified for protein *s*. *C*_*x,s,i*_ denotes the intensity of protein *s* for subject *i* from group *x* (“1” for the Case group and “2” for the Control group), and *d* is a constant representing the limit of detection. The univariate likelihood can be rewritten as:

(1) $$L\left(\Theta_{s}\right)=\prod_{i=1}^{n}f\left(C_{1,s,i}|\mu_{s},\kappa_{s},\sigma_{s}^{2},d\right)\;\prod_{j=1}^{m}f\left(C_{2,s,j}|\mu_{s},\delta_{s},\kappa_{s},\tau_{s},\sigma_{s}^{2},d\right)$$

The likelihood for each individual protein intensity, *C*_*x,s,i*_ is calculated by the univariate likelihood model proposed previously;

(2) $$f\left(C_{x,s,i}|\mu_{x},\sigma_{x}^{2},\rho_{x},d\right)=\{\begin{array}{cc} \left(1-\rho_{x}\right)+\rho_{x}\int_{-\infty }^{d}\frac{1}{\sigma_{x}\sqrt{2\pi }}\exp\left(-\frac{{\left(y-\mu_{x}\right)}^{2}}{2\sigma_{x}^{2}}\right)\;dy & i\text{f}\;C_{x,s,i}<d\\ \frac{\rho_{x}}{\lambda }\left[\frac{1}{\sigma_{x}\sqrt{2\pi }}\exp\left(-\frac{{\left(C_{x,s,i}-\mu_{x}\right)}^{2}}{2\sigma_{x}^{2}}\right)\right] & \text{otherwise} \end{array}$$

and *λ* is the scaling factor to ensure the truncated normal distribution integrates to one:

(3) $$\lambda =\int_{d}^{v}\frac{1}{\sigma_{x}\sqrt{2\pi }}\exp\left(-\frac{{\left(y-\mu_{x}\right)}^{2}}{2\sigma_{x}^{2}}\right)\;dy$$

where *d* is the limit of detection and *ν* is the maximum expression value.

Briefly, the univariate model allows for two cases in Equation 1:

(1) When the intensity, *C*_*x,s,i*_ is less than the level of detection, the the likelihood function reflects a mixture of the possibilities that either the protein was not expressed (i.e., *1 – ρ*_*x*_, where *ρ*_*x*_ is the probability of expression), or that the protein was expressed but fell below the level of detection (the second term on the right hand side of the first row, in Equation 2).

(2) When the intensity is greater than the level of detection, the likelihood function is given by a truncated normal distribution, with the lower tail truncated at *d*, the level of detection (second row of Equation 2).

The joint likelihood for all proteins at the local layer is the product of the likelihood for each individual protein and can be calculated as:

(4) $$L\left(\Theta_{L}\right)=\prod_{s=1}^{S}L\left(\Theta_{S}\right)$$

where *L(Θ*_*L*_*)* is the likelihood for all proteins at the local layer and *S* is the total number of proteins in the 2D PAGE experiment.

### The global layer

The global layer ties all the parameters in the local layer together. All mean expression intensities for the individual proteins from the Control group are assumed to be normally distributed with mean *u*_*g*_ and standard deviation *σ*_*g*_. The likelihood function is:

(5) $$f\left(\mu_{s}|\mu_{g},\sigma_{g}\right)=\frac{1}{\sigma_{g}\sqrt{2\pi }}\exp\left(-\frac{{\left(\mu_{s}-\mu_{g}\right)}^{2}}{2\sigma_{g}^{2}}\right)$$

All proteins are assumed to have the same standard deviation of expression intensities (measured on the log scale), which is calculated by multiplying *σ*_*g*_ by the spot standard deviation scalar parameter *ψ*. Therefore the spot standard deviation *σ*_*s*_ *= ψσ*_*g*_ is used to calculate the likelihood for each spot in the local layer. This allows the model to efficiently estimate the spot standard deviation and explore the potential relationship between *σ*_*s*_ and *σ*_*g*_.

To model the distribution of mean expression intensities for proteins from the Case group, we use *δ*_*s*_ as the difference between mean expression intensities between Case and Control groups. Each 2D PAGE experiment detects a large number of proteins (800 ∼ 1200) and the difference between two mean expression intensities *δ*_*s*_ is generally close to zero for most of the proteins. An appropriate distribution for *δ*_*s*_ is the exponential distribution, which has a peak at 0. However, since there can be both negative and positive values of *δ*_*s*_, we use a modified Laplace distribution centered at zero. The Laplace distribution is essentially two exponential distributions, decaying symmetrically in both directions, from a mean of zero. The modification we make is to allow each side of the Laplace distribution to be weighted differently. This allows different numbers of Case group proteins to be up regulated (positive values of *δ*_*s*_) or down regulated (negative values of *δ*_*s*_). The proportion of up-regulated proteins is *ϕ*_*δ*_, and is bounded between zero and one. Therefore the proportion of down-regulated proteins can be calculated as *1-ϕ*_*δ*_. The likelihood function for *δ*_*s*_ is:

(6) $$f\left(\delta_{s}|\lambda_{\delta },\varphi_{\delta }\right)=\{\begin{array}{cc} \left(1-\varphi_{\delta }\right)\lambda_{\delta }e^{-\lambda_{\delta }-\delta_{s}} & \delta_{s}<0\\ \;\varphi_{\delta }\lambda_{\delta }e^{-\lambda_{\delta }\delta_{s}} & \delta_{s}\geq 0 \end{array}$$

Both parameters relating to the probability of expression follow normal distributions: at the global layer, the values of *κ*_*s*_ (the probability of individual protein expression in the Control group) are random variables drawn from a normal distribution with mean *μ*_*κ*_ and standard deviation *σ*_*κ*_. Similarly, the parameters specifying the expression probabilities in the control and case groups, *κ*_*s*_ and *τ*_*s*_, are random variables of a normal distribution with mean *μ*_*τ*_ and standard deviation *σ*_*τ*_. The likelihood equations for these parameters are:

(7) $$f\left(\kappa_{s}|\mu_{\kappa },\sigma_{\kappa }\right)=\frac{1}{\sigma_{\kappa }\sqrt{2\pi }}\exp\left(-\frac{{\left(\kappa_{s}-\mu_{\kappa }\right)}^{2}}{2\sigma_{\kappa }^{2}}\right)$$

and

(8) $$f\left(\tau_{s}|\mu_{\tau },\sigma_{\tau }\right)=\frac{1}{\sigma_{\tau }\sqrt{2\pi }}\exp\left(-\frac{{\left(\tau_{s}-\mu_{\tau }\right)}^{2}}{2\sigma_{\tau }^{2}}\right)$$

In total, there are nine parameters at the global layer, and the marginal likelihood for the local parameters can be expressed as:

(9) $$\prod_{s=1}^{S}f\left(\mu_{s},\delta_{s},\kappa_{s},\tau_{s}|\mu_{g},\delta_{g},\psi ,\lambda_{g},\varphi_{\delta },\mu_{\kappa },\delta_{\kappa },\mu_{\tau },\delta_{\tau }\right)$$

### Markov Chain Monte Carlo (MCMC)

#### Bayesian inference and the Metropolis-Hastings algorithm

Bayesian inference recovers the degree of belief in the values of parameters by combining information from the data and *a priori* knowledge of the distribution of model parameters. The result is a posterior distribution *p(θ|D)*, which is often expressed as:

(10) $$p\left(\theta |D\right)\propto p\left(D|\theta \right)p\left(\theta \right)$$

Here, *p(D|θ)* denotes the likelihood function, and *p(θ)* is the prior distribution of the parameter set *θ*. The posterior distribution *p(θ|D)* summarizes the degree of belief in *θ*, based on the observed data, *D,* and prior knowledge of the parameter set.

For complex analyses, including the estimation of parameters in many mixture models, it is often difficult to obtain the posterior distribution directly. Markov chain Monte Carlo (MCMC) integration is a computationally tractable and commonly used solution to the problem. It is an iterative procedure which attempts to recover the posterior distribution by sampling the permissible parameter space. One common implementation of MCMC uses the Metropolis-Hasting algorithm [13,14], which can be described by the following steps.

Step 1: Begin with initial state Θ.

Step 2: Make a small change to the parameter *θ*^*i*^ to θ* according to a proposal distribution q(θ*|θ^*i*^*)*.

Step 3: Calculate the acceptance ratio α, using the following formula:

(11) $$\alpha =\min\left\{1,\;\frac{f\left(\Theta^{*}|d\right)q\left(\theta^{i}|\theta^{*}\right)}{f\left(\Theta^{i}|d\right)q\left(\theta^{*}|\theta^{i}\right)}\right\}$$

Generate μ from *U(0, 1)* and accept *θ*^*i+1*^ = θ^*^ if *μ < α*.

Otherwise *θ*^*i+1*^ *= θ*^*i*^.

Step 4: Set *i = i + 1* and repeat Step 1.

The algorithm is repeated until the Markov chain is sampling from the target distribution, typically the (joint) posterior distribution of the parameter(s).

When the Markov chain reaches the stationary or equilibrium distribution, the 95% highest posterior density (HPD) region for the marginal posterior distribution for each parameter can be calculated. The 95% HPD region consists of the smallest collection of potential parameter values such that the marginal posterior probability of the parameter falling into this region is at least 95%.

### Prior and proposal distributions

Bayesian inference requires a choice of prior distributions that reasonably characterize the uncertainty in the parameter values before new data are added, or that are based on distributional information that may be gleaned from previous analyses [15]. Here, we have chosen prior distributions using the former approach, although the “reasonableness” (or otherwise) of these distributions have been loosely assessed against previously obtained data (Table 1). The method we describe can, of course, be used for any set of prior distributions, and the software we developed can be modified to accommodate alternative priors; we recommend, however, that users choose prior distributions that suit their specific experimental design.

**Table 1:** List of prior distributions used in the global model

**Global parameter*θ***^***i***^	**Prior distribution*p(θ***^***i***^***)***
*μ*_*g*_	*Normal ∼ (μ = −3,σ = 5)*
*σ*_*g*_	*Γ*^*-1*^*(shape = 0.001, rate = 0.001)*
*ψ*	*Uniform ∼ (0.001, 2)*
*λ*_*δ*_	*Exponential ∼ (λ = 1)*
*ϕ*_*δ*_	*Beta ∼ (alpha = 2,beta = 2)*
*μ*_*κ*_	*Normal(μ = 0,σ = 3)*
*σ*_*κ*_^*2*^	*Γ*^*-1*^ *∼ (shape = 0.001,rate = 0.001)*
*μ*_*τ*_	*Normal ∼ (μ = 0,σ = 3)*
*σ*_*τ*_^*2*^	*Γ*^*-1*^ *∼ (shape = 0.001,rate = 0.001)*

For the global mean expression intensity *μ*_*g*_, we used a normal distribution centered at −3 with a standard deviation of 5 as the prior. The prior is centered at −3 as the data are log-transformed relative protein expression intensities. If a gel has 1000 proteins with identical expression intensities, then the mean relative percentage volume expression will be 0.1 for each protein, which is ∼ −3.3 when log_2_-transformed. However, since we do not know the true mean volume, a relatively large standard deviation was assigned to the prior distribution of relative expression intensities. There was insufficient information to provide a good estimate of the prior distribution for the global standard deviation *σ*_*g*_, therefore a relatively flat inverse-gamma prior *σ*_*g*_ *∼ Γ*^*-1*^*(0.001,0.001)* was used [16].

The modified Laplace distribution is used to model the difference between two mean expression intensities. This distribution has two parameters: *λ*_*δ*_ is the rate for the exponential distribution component, and *ϕ*_*δ*_ is the proportion of up-regulated proteins. The rate parameter has an exponential prior of *λ*_*δ*_ *∼ Exp (1)*. The proportion of up-regulated proteins *ϕ*_*δ*_ is bounded between 0 and 1. If there is approximately an equal number of up- and down- regulated proteins then the value of *ϕ*_*δ*_ will be close to 0.5. Therefore the density function for the prior should peak around 0.5 and decrease as *ϕ*_*δ*_ moves toward 0 or 1, thus, a Beta (2,2) distribution was used as the prior for *ϕ*_*δ*_.

The means for both the probability of expression in the Control group, *μ*_*κ*_, and the difference between probabilities of expression between the two groups, *μ*_*τ*_, have more stringent priors. A normal distribution centered at 0 with a standard deviation of 3 is used for both parameters. Under the reparameterisation procedures described earlier, $p_{0}=\frac{\exp\left(\kappa \right)}{1+\exp\left(\kappa \right)}$ and $p_{1}=\frac{\exp\left(\kappa +\lambda \right)}{1+\exp\left(\kappa +\lambda \right)}$, if the probabilities of expression for the control group are given by *ρ*_*0*_ = 0.95, this would correspond to *κ ∼2.94*. We believe that it is unnecessary to distinguish the probability of expression between 0.95 and 1 because the difference is unlikely to be biologically significant. Therefore a relatively small standard deviation was assigned to the prior distribution to avoid *κ*_*s*_ or *τ*_*s*_ moving towards very large values. Consequently, this also prevents false positive results which may occur when the model attempts to distinguish the difference between probabilities of expression beyond 0.95.

A proposal distribution, *q(θ),* was used to generate a candidate value θ* based on the current parameter value *θ*^*i*^ with the probability q(θ*|θ^*i*^*)*. The proposal distributions used in this paper are also given in Table 2, and are typical for the types of parameters in our model. The following describes the rationale for the use of non-standard proposal distributions for a subset of parameters.

**Table 2:** List of proposal distributions for both global and local parameters

**Global parameter*θ***^***i***^	Proposal distributionq(θ*|θ^***i***^***)***
*μ*_*g*_	*Truncated-Normal ∼ (μ = μ*_*g*_*, lower = d, upper = log*_*2*_*(100))*
*σ*_*g*_	*Truncated-Normal ∼ (μ = σ*_*g*_*, lower = 0.01)*
*ψ*	*Truncated-Normal ∼ (μ = ψ, lower = 0.001, upper = 2)*
*λ*_*δ*_	*Truncated-Normal ∼ (μ = λ*_*δ*_*, lower = 0.01)*
*ϕ*_*δ*_	*ϕ*_*δ*_*' = Normal ∼ (μ = ln[ϕ*_*δ*_*/(1-ϕ*_*δ*_*)]), ϕ*_δ_* = exp(ϕ_*δ*_*')/[1 + exp(ϕ*_*δ*_*')]*
*μ*_*κ*_	*Normal ∼ (μ = μ*_*κ*_*)*
*σ*_*κ*_	*Truncated-Normal ∼ (μ = σ*_*κ*_*, lower = 0.01)*
*μ*_*τ*_	*Normal ∼ (μ = μ*_*τ*_*)*
*σ*_*τ*_	*Truncated-Normal ∼ (μ = σ*_*τ*_*, lower = 0.01)*
**Local parameter *θ***^***i***^	Proposal distribution q(θ*|θ^***i***^***)***
*μ*_*s*_	*Normal ∼ (μ = μ*_*s*_*)*
*δ*_*s*_	*Normal ∼ (μ = δ*_*s*_*)*
*κ*_*s*_	*Normal ∼ (μ = κ*_*s*_*)*
*τ*_*s*_	*Normal ∼ (μ = τ*_*s*_*)*

*d* = limit of detection. The standard deviation for all proposal distributions are controlled by the tuning parameters.

The proportion of up-regulated proteins *ϕ*_*δ*_ was bounded between 0 and 1. Therefore a logit transformation was applied to *ϕ*_*δ*_ to obtain a value without boundaries *logit(ϕ*_*δ*_*) = ϕ*_*δ*_*/(1-ϕ*_*δ*_*)*. A normal distribution with mean set to *logit(ϕ*_*δ*_*)* was then used to propose a new value *ϕ*_*δ*_*'*. Finally, an inverse-logit transformation was applied to *ϕ*_*δ*_*'* to obtain the candidate value *ϕ*_δ_* which is always between 0 and 1.

The global standard deviation, *σ*_*g*_, the rate parameter for the exponential distribution, *λ*_*δ*_, the standard deviation for the probabilities of expression, *σ*_*κ*_, and the standard deviation for the difference in the probabilities of expression, *σ*_*τ*_, all have the same proposal distributions, a truncated normal distribution with lower bound set to 0.01 and no upper bound. The theoretical lower limit for these values is 0, but 0.01 was used for two reasons. The first was that these values were extremely unlikely to be less than 0.01 for any 2D PAGE experiments. Hundreds of different proteins were separated in each 2D PAGE experiment and it is unlikely for all the proteins to have very similar means and probabilities of expression. The mean of the exponential distribution is *1/λ*, and the theoretical maximum intensity for a protein on 2D PAGE is *log*_*2*_*(100) ≈ 6.64*. Therefore we expect *λ*_*δ*_ to be greater than 0.01 because the mean value for *δ*_*s*_ (the difference between two mean expression intensities) is unlikely to be greater than 100. The second reason was to prevent floating point underflow when computing extremely small likelihood values when the standard deviation approaches 0.

### Adaptive MCMC

Since MCMC is a technique that relies on a stochastic perturbation to the current state to generate the next state in a chain, the states are autocorrelated. Depending on the proposal distributions used, there is a possibility for states to persist in a part of parameter space, and mix poorly. We used three different techniques to improve the mixing of the Markov chain: tuning parameters, block updating and parameter expansion.

Roberts et al. [17] suggest that for a single dimension problem the optimal acceptance ratio should be 0.43, and 0.234 for higher dimension problems. During each iteration, proposed values are recorded regardless of whether they are accepted or not. The acceptance rate is calculated and proposal distribution parameters updated according to the following formula,

(12) $$\sigma_{\mathrm{new}}=\frac{\sigma_{\mathrm{cur}}\Phi^{-1}\left(\frac{\rho_{\mathrm{opt}}}{2}\right)}{\Phi^{-1}\left(\frac{\rho_{\mathrm{cur}}}{2}\right)}$$

where *σ*_*new*_ is the standard deviation of the new proposal distribution and *σ*_*cur*_ is the standard deviation of the current proposal distribution. *ρ*_*opt*_ is the optimal acceptance ratio, *ρ*_*cur*_ the current acceptance ratio, and *Φ*^*-1*^ is the inverse CDF of a standard normal distribution. If the acceptance ratio is higher than the optimal acceptance ratio, then the standard deviation for the proposal distribution is increased to lower the acceptance ratio and vice versa [18]. The standard deviation *σ*_*new*_ is updated once every 500 iterations and the current acceptance ratio *ρ*_*cur*_ is averaged over 3000 iterations.

The second technique is block updating, which was used to reduce the autocorrelation for related parameters [19]. A block is created by grouping two or more related variables and updating them simultaneously. If two variables are in the same block, then two values will be proposed for each iteration of the chain. Only one Metropolis-Hasting ratio will be calculated, and both values are then either jointly accepted or rejected. For example, if two parameters *θ*_*1*_ and *θ*_*2*_ are paired together, then the joint acceptance ratio is calculated by:

(13) $$\alpha =\min\left\{1,\frac{f\left(\Theta^{*}|d\right)q\left(\theta_{1}^{i}|\theta_{1}^{*}\right)q\left(\theta_{2}^{i}|\theta_{2}^{*}\right)}{f\left(\Theta^{i}|d\right)q\left(\theta_{1}^{*}|\theta_{1}^{i}\right)q\left(\theta_{2}^{*}|\theta_{2}^{i}\right)}\right\}$$

At the local layer, we paired *μ*_*s*_ and *δ*_*s*_ together and *κ*_*s*_ and *τ*_*s*_ together. At the global level, we paired *μ*_*g*_ and *σ*_*g*_ together, *λ*_*δ*_ and *ϕ*_*δ*_ together, *μ*_*κ*_ and *σ*_*κ*_ together and, *μ*_*τ*_ and *σ*_*τ*_ together. Sometimes the variance parameter was not able to move freely, especially when it approached zero, resulting in poor mixing. The introduction of an additional parameter which links mean and variance together can potentially reduce this issue [20]. This is termed “parameter expansion” and it was implemented here to reduce this problem.

Three parameters were added to the global likelihood model. The term *α*_*μ*_ was added to link global mean *μ*_*g*_ and standard deviation *σ*_*g*_, and was calculated in the following way:

(14) $$\mu_{g}=\alpha_{\mu }\mu_{g'}\;\delta_{g}^{2}=\alpha_{\mu }^{2}\sigma_{g'}^{2}$$

Within each iteration, instead of one block updating which paired *μ*_*g*_ and *σ*_*g*_^*2*^ together, two block updating was used after parameter expansion was implemented. One block updates paired *μ*_*g*_*'* and *σ*_*g*_^*2*^*'* together, and the other one updates *α*_*μ*_. The other two parameters are *α*_*κ*_ which links *μ*_*κ*_ and *σ*_*κ*_^*2*^ together, and *α*_*τ*_ which links *μ*_*τ*_ and *σ*_*τ*_^*2*^ together. These two parameters were implemented and updated in the same way as *α*_*μ*_. All three parameters had a uniform prior between 0.01 and 10, and a truncated normal distribution was used as their proposal distribution (Table 3). The mean of the proposal distribution is the current parameter value and the standard deviation was controlled by the tuning parameter descried in this section.

**Table 3:** Prior and proposal distributions used for the parameters introduced in the parameter expansions

**Global parameter*θ***^***i***^	**Prior distribution*p(θ***^***i***^***)***	Proposal distributionq(θ*|θ^***i***^***)***
*α*_*μ*_	*Uniform ∼ (0.01,10)*	*Truncated-Normal ∼ (μ = α*_*μ*_*, lower = 0.01)*
*α*_*κ*_	*Uniform ∼ (0.01,10)*	*Truncated-Normal ∼ (μ = α*_*κ*_*, lower = 0.01)*
*α*_*τ*_	*Uniform ∼ (0.01,10)*	*Truncated-Normal ∼ (μ = α*_*τ*_*, lower = 0.01)*

With the combination of block updating and parameter expansion, there were twelve parameters, including nine parameters from the likelihood model and three tuning parameters (*α*) described above. These parameters were grouped and updated in eight different blocks.

### Simulation analysis

**Figure 1:**
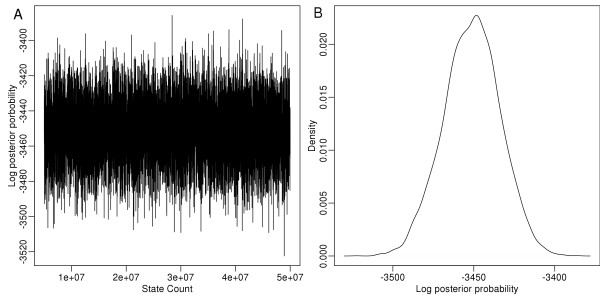
The trace plot (A) and density plot (B) for the log posterior probability from Simulation 1.

In order to evaluate the global model, we simulated 2D-PAGE data based on studies described in our previous paper [9] and compared the results against those obtained using the LRT proposed therein. A set of global distributions and global parameters were described above and predefined for each simulation. All individual local parameters for each protein were drawn from the global distributions. The probability of expression parameters for each individual protein determined whether a protein was expressed. The expression intensities for an expressed protein were drawn from a normal distribution with an individual protein mean. The limit of detection was set to – 8.67, and any simulated value below this threshold was treated as missing data. One hundred proteins were simulated because of the amount of time required for a MCMC chain to converge (approximately 20 ∼ 24 hours for 100 proteins). The MCMC algorithm for the global likelihood model was implemented using Java. Thinning was used to reduce the autocorrelation and we sampled the states every 1000 iterations. The MCMC chain ran for 50 million iterations and we manually inspected the trace plot of the posterior probability from multiple runs to check for any inconsistencies. The first 10% of the data was discarded as burn-in, to allow the Markov chain to reach the target distribution. The Effective Sample Size (ESS) calculated for every parameter. The ESS is the effective number of “independent” samples from the Markov chain. All the ESS were calculated using Tracer (http://beast.bio.ed.ac.uk/Tracer) [21]; in our analyses, the minimum ESS was always greater than 1000. The trace plot and density plot for the log posterior distribution from Simulation 1 are shown in Figure 1.

Once we were confident that the Markov chain was sampling the target distribution, the 95% highest HPD for *δ*_*s*_ and *τ*_*s*_ was calculated. The local parameter *δ*_*s*_ and *τ*_*s*_ represent the differences in mean expression intensities between Case and Control groups and the probability of expression, respectively. There are three scenarios whereby a protein may be classified as statistically differentially expressed: 1) If the 95% HPD for *δ*_*s*_ does not include zero, 2) if the 95% HPD for *τ*_*s*_ does not include zero, or 3) if the 95% HPDs for both parameters do not include zero.

### Simulation 1. Simulation based on a real experiment

100 differentially expressed proteins, with each protein having different parameter values, were drawn from a global distribution with the following parameters: the mean expression intensities for the control group followed a normal distribution with a mean of −5 and a standard deviation of 1. The standard deviation for each individual protein was 0.7. The difference between mean expression intensities was drawn from a modified Laplace distribution (described in the global layer section) with *λ*_*δ*_ = 0.5 and *ϕ*_*δ*_ = 0.5. The parameter associated with the probability of expression, *κ*_*s*_ was drawn from a normal distribution with a mean of 1 and a standard deviation of 1, and *τ*_*s*_ was drawn from a normal distribution with a mean of 0 and a standard deviation of 2.

### Simulation 2. Varying the global distribution of the probabilities of expression

The second simulation was similar to Simulation 1, except that the values of *κ*_*s*_ were no longer assumed to follow a normal distribution. Instead, for each protein, *κ*_*s*_ was drawn from a uniform distribution between −1 and 3, and *τ*_*s*_ was drawn from a uniform distribution between −2 and 2. All other global parameters were identical to those specified in Simulation 1.

### Simulation 3. A smaller gap between mean expression intensities and different distributions for the probabilities of expression

In the previous two simulations, *λ*_*δ*_ for the modified Laplace distribution was set to 0.5, which corresponds to a difference between two mean expression intensities of 2. In Simulation 3, the difference between two mean expression intensities was set to 1.5 times the protein standard deviation, which corresponds to *λ*_*δ*_ ≈ 0.66. This was done because results from our previous study showed that LRT had a reasonable performance when the difference between the two mean expression intensities was approximately 1.5 times the standard deviation or higher. This simulation also tested the difference between two probabilities of expression when drawn from two different distributions. For each individual protein, *κ*_*s*_ was still drawn from a normal distribution with mean 1 and a standard deviation of 0.25, but *τ*_*s*_ was divided into two groups. Half of the proteins were simulated from a normal distribution with mean −3 and standard deviation of 0.25; the other half were simulated from a normal distribution with mean 2 and standard deviation of 0.25. Note that we assigned a relatively small standard deviation to these distributions to obtain two non-overlapping normal distributions. This extreme scenario is used to test the flexibility of the Bayesian model. All other global parameters were identical to Simulation 1.

### Simulation 4. Estimating the false positive rate

This simulation attempted to investigate the number of proteins falsely classified as differentially expressed when there was no difference between two groups. The difference between local mean expression intensities *δ*_*s*_ and the difference between local probabilities of expression *τ*_*s*_ were fixed at 0 for all proteins. All other global parameters were identical to Simulation 1. This setting makes two groups identical and allows us to estimate the false positive rate of this model.

### Application of model to 2D PAGE data

We also applied the global model to a 2D PAGE experiment reported previously by Wu et al [9] in which we selected differentially expressed spots based on a likelihood ratio test This experiment contained 24 individuals, with one gel per individual. Eight hundred and three spots were detected and matched using commercial software.

## Results and discussions

Both the global model and the LRT previously defined in Wu et al (2009) were applied to the three simulations.

### Simulation 1. Simulation based on a real experiment

The mean and the 95% HPD were calculated from the marginal posterior distribution for all the global parameters and summarized in Table 4. The true values for several global parameters were very accurately recovered: the mean values recovered were very close to the true values, for example, the recovered mean for *μ*_*g*_ was −4.8 (true value was −5), and the recovered mean for *σ*_*g*_ was 1.06 (true value = 1). The 95% HPD for most of the global parameters included the true values, for example, the recovered mean for *μ*_*κ*_ was 0.89 with the 95% HPD between 0.66 and 1.15 while the true value was 1, the recovered mean for *μ*_*τ*_, was −0.22 with the 95% HPD between −0.75 and 0.38, while the true value was 0.

**Table 4:** Summary of the global parameters for simulation 1, which is based on a real 2D PAGE experiment

**Global parameter**	**Mean from MCMC**	**Lower 95% HPD**	**Upper 95% HPD**	**True value**
*μ*_*g*_	−4.8	−5.02	−4.59	−5
*σ*_*g*_	1.06	0.92	1.22	1
*ψ*	0.65	0.56	0.75	0.7
*λ*_*δ*_	0.57	0.46	0.69	0.5
*ϕ*_*δ*_	0.57	0.45	0.67	0.5
*μ*_*κ*_	0.89	0.66	1.15	1
*σ*_*κ*_	1.00	0.78	1.24	1
*μ*_*τ*_	−0.22	−0.75	0.38	0
*σ*_*τ*_	2.48	1.93	3.08	2

**Figure 2:**
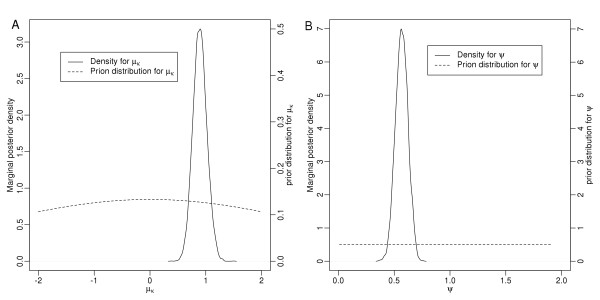
**Marginal posterior density and prior distribution for the global parameter (A) μ**_**κ**_**and (B) ψ**.

Figure 2 shows the marginal posterior density and prior distributions for the global parameters *μ*_*κ*_ and *ψ*. The marginal posterior distributions were substantially different from the prior distributions used in the model. The approach of plotting the posterior distribution against that of the prior is valuable, because it shows that the extent to which the addition of new data reduces the uncertainty in the model. The 95% HPDs were also calculated for all the local parameters *δ*_*s*_ and *τ*_*s*_, and 85 spots were classified as differentially expressed. The LRT was applied to the same dataset and only 71 spots were classified as differentially expressed.

**Figure 3:**
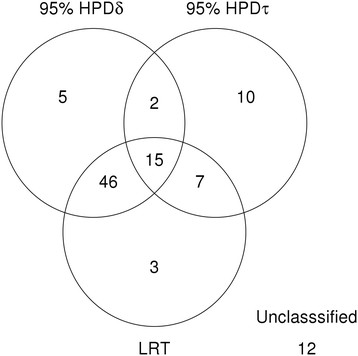
**Number of proteins classified as differentially expressed using each method in Simulation 1** .

All but three of the 71 spots identified using the LRT were also identified using the method reported here. There were 12 differentially expressed proteins that were not correctly classified by both methods. The Venn diagram in Figure 3 summarizes the differentially expressed spots classified by each method.

The recovered mean for the proportion of up-regulated proteins *ϕ*_*δ*_ was 0.57 with the 95% HPD between 0.45 and 0.67 (the true value is 0.5). This implied that 57% of the spots were considered as up-regulated, that is, the mean expression intensity for the case group was higher than the control group. Nevertheless, this does not represent the proportion of statistically classified differentially expressed proteins because the statistical classification of up- or down regulation depends on whether the 95% HPD of *δ*_*s*_ for each protein includes zero. Under this criterion, there were 38 spots that were (statistically) classified as up-regulated and 30 spots that were (statistically) classified as down-regulated.

### Simulation 2. The effect of the underlying global distribution on the probabilities of expression

The mean and the 95% HPD were calculated from the marginal posterior distribution for all the global parameters and are summarized in Table 5. The mean for the four parameters *μ*_*g*_, *σ*_*g*_, *ψ*, and *ϕ*_*δ*_, were very close to the true value, with the absolute difference less than 0.1. The 95% HPD interval for *λ*_*δ*_ (0.5 and 0.79) also included the true value 0.7.

**Table 5:** Summary of the global parameters for simulation 2 where the probabilities of expression were drawn from uniform distributions

**Global parameter**	**Mean from MCMC**	**Lower 95% HPD**	**Upper 95% HPD**	**True value**
*μ*_*g*_	−5.00	−5.23	−4.79	−5
*σ*_*g*_	1.08	0.93	1.23	1
*ψ*	0.69	0.59	0.79	0.7
*λ*_*δ*_	0.62	0.50	0.75	0.5
*ϕ*_*δ*_	0.54	0.43	0.65	0.5
*μ*_*κ*_	0.99	0.70	1.3	κ ∼ Uniform(−1,3)
*σ*_*κ*_	1.29	1.03	1.56	κ ∼ Uniform(−1,3)
*μ*_*τ*_	−0.23	−0.67	0.22	τ ∼ Uniform(−2,2)
*σ*_*τ*_	1.84	1.41	2.29	τ ∼ Uniform(−2,2)

**Figure 4:**
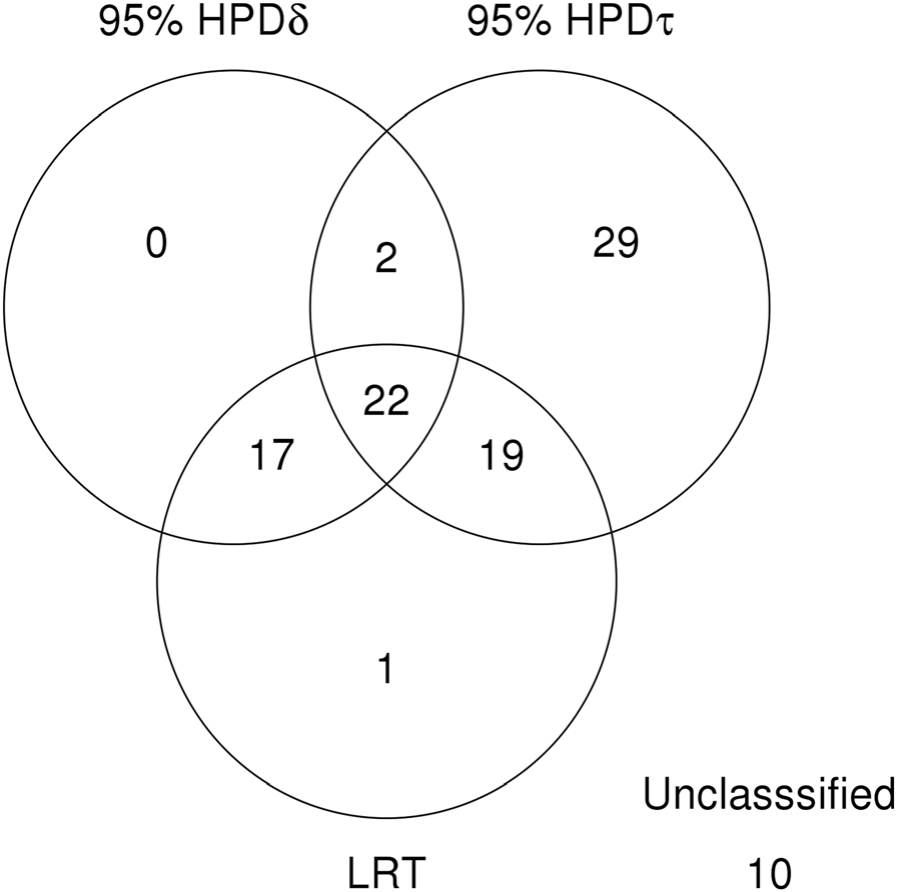
**Number of proteins classified as differentially expressed using each method in Simulation 2** .

Figure 4 summarizes the number of proteins classified as statistically differentially expressed under each category. The LRT classified 59 spots as differentially expressed and the global likelihood model classified 89 proteins. Only one of the spot identified by the LRT was not identified by the model reported here.

**Figure 5:**
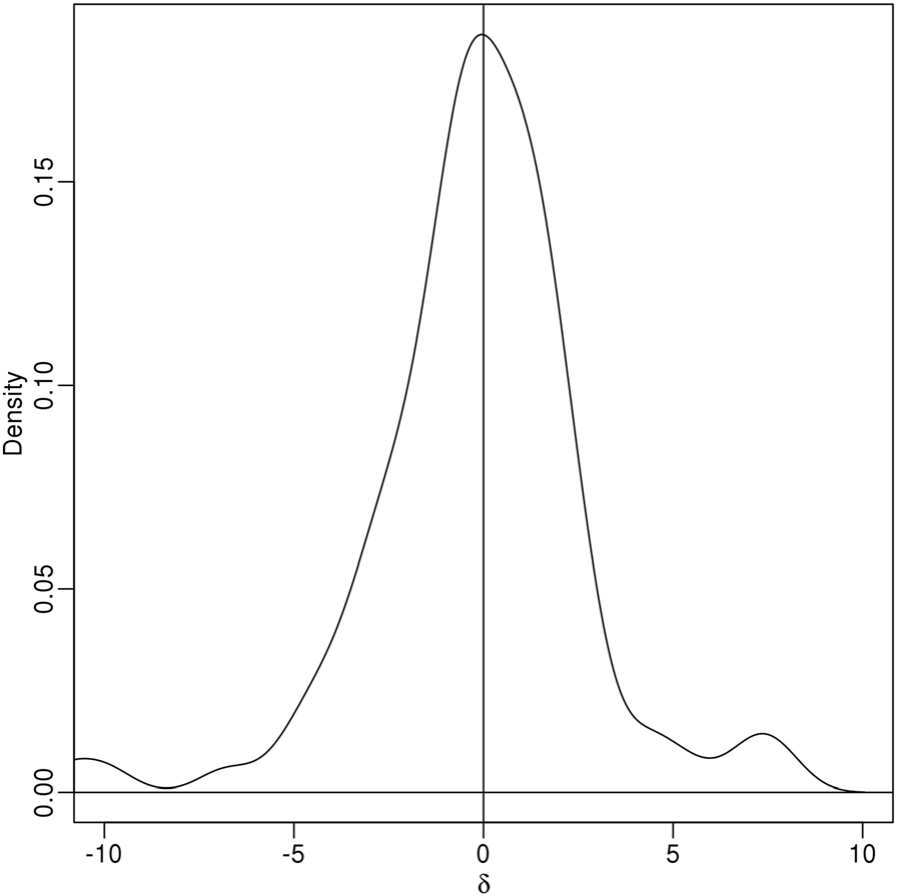
**Density for the true values of 100 local parameters δ**_**s**_**.** This shows that the distributions for values of δ_s_ greater and less than 0 were approximately symmetrical.

There were 38 spots classified as statistically up-regulated and 25 spots classified as statistically down-regulated. By examining the true values of 100 local parameters, *δ*_*s*_, the distributions of *δ*_*s*_ have heavier tail for values greater than 0 then values less than 0 (there are more *δ*_*s*_ greater than 5 then less than −5) (Figure 5)hence the there are more spots are statistically classified as up-regulated than down-regulated.

### Simulation 3. Smaller difference between mean expression intensities and alternative distributions for the probabilities of expression

**Figure 6:**
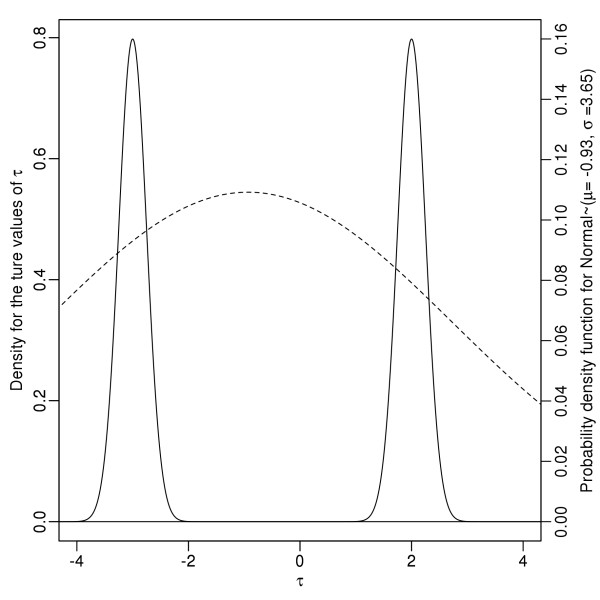
**The density plot for the parameters τ and the global distrubituon recovered by the model.** The probability density function Normal ∼ (μ_τ_ = 0.5,σ_τ_ = 3.38) where μ_τ_ and σ_τ_ were recovered by the global model.

The mean and the 95% HPD were calculated from the marginal posterior distribution for all the global parameters and are summarized in Table 6. The 95% HPD intervals for most of the parameters included the true values used to simulate the dataset. The two exceptions were *μ*_*τ*_ and *σ*_*τ*_, which were parameters where recovery of the true underlying distributions was not expected since the local parameters *τ*_*s*_ were simulated from two distinct normal distributions that did not overlap. Therefore a single normal distribution was not expected to recover the true values. Figure 6 shows the density plot for the 100 local parameters *τ,* and the probability density function for the normal distribution with parameters *μ*_*τ*_ and *σ*_*τ*_ recovered by the global model. The global model adjusted to this change in data by increasing the value of *σ*_*τ*_ to a large number with a mean value of 3.65 and 95% HPD interval between 2.89 and 4.46. This effectively created a very wide normal distribution which was used to ensure all the *τ*_*s*_ drawn from both underlying normal distributions would have similar likelihoods. This demonstrates that the global likelihood model is very robust and is able to adapt to different distributions even if the local parameters were not drawn from a single distribution.

**Table 6:** Summary of the global parameters for simulation 3 where the difference between two probability of expressions were drawn from two normal distributions

**Global parameter**	**Mean from MCMC**	**Lower 95% HPD**	**Upper 95% HPD**	**True value**
*μ*_*g*_	−5.18	−5.39	−4.95	−5
*σ*_*g*_	1.13	0.98	1.29	1
*ψ*	0.64	0.55	0.73	0.7
*λ*_*δ*_	0.73	0.57	0.89	0.7
*ϕ*_*δ*_	0.47	0.35	0.60	0.5
*μ*_*κ*_	0.98	0.83	1.12	1
*σ*_*κ*_	0.28	0.11	0.46	0.25
*μ*_*τ*_	−0.93	−1.76	−0.16	*
*σ*_*τ*_	3.65	2.89	4.46	*

* 50% τ ∼ Normal (−3, 0.25), 50% τ ∼ Normal (2, 0.25).

**Figure 7:**
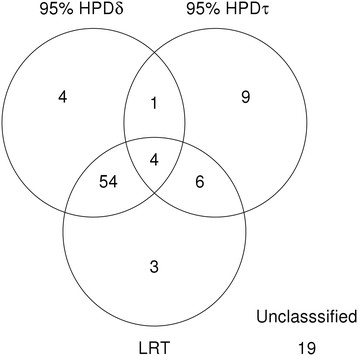
Number of proteins classified as differentially expressed using each method in Simulation 3.

Figure 7 summarizes the number of proteins classified as differentially expressed under each category. The LRT classified 67 spots as differentially expressed compared to 78 in the global Bayesian model. The LRT only picked up three spots that were missed by the method described here.

### Simulation 4. Estimating the false positive rate

The 95% HPDs were calculated for all the local parameters *δ*_*s*_ and *τ*_*s*_, and all the HPD intervals contained zero. This implied that none of the proteins were classified as differentially expressed. The simulations were repeated with 18 and 24 gels in each group while all other parameters remained the same. Once again, in these further simulations none of the proteins was classified as differentially expressed. This demonstrates that the model we propose here has a very low false positive rate.

### 2D PAGE Example

**Figure 8:**
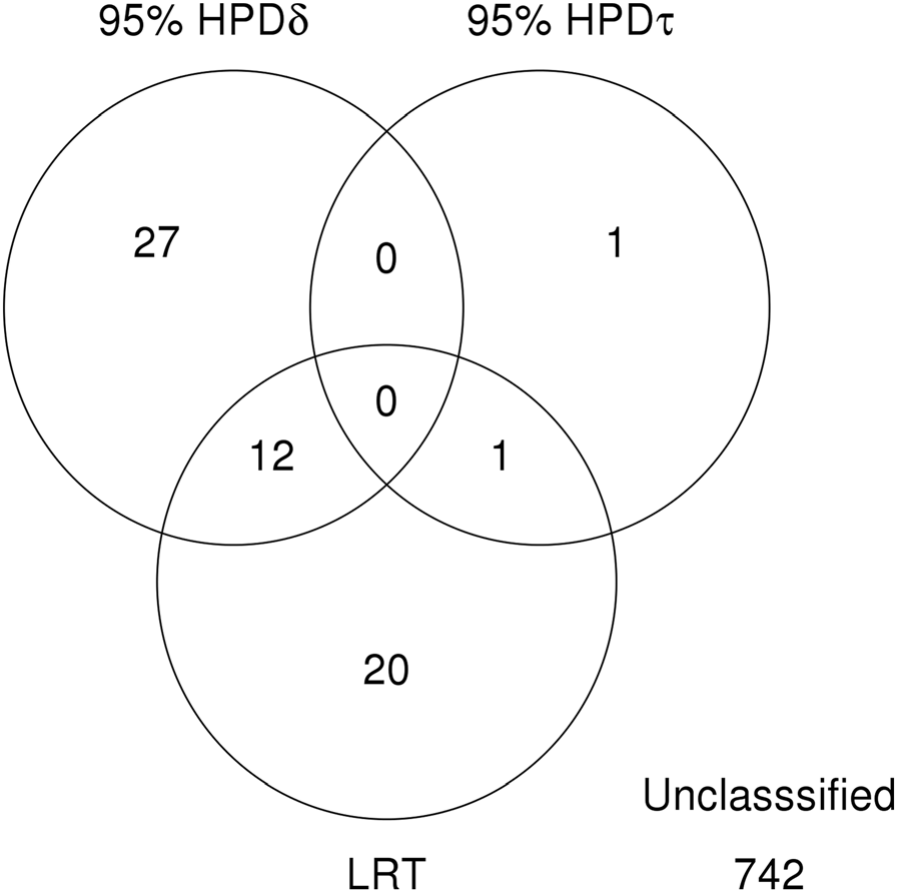
Number of proteins classified as differentially expressed using each method in 2D PAGE data.

Figure 8 summarizes the number of proteins classified as differentially expressed using the MCMC procedure described here (separated according to whether the expression intensity, *δ*, or probability of expression, *τ*, differed between Case and Control), and the previously described LRT procedure [9]. The univariate LRT classified 33 spot as differentially expressed compared to 41 in the global Bayesian model. However, several spots classified using the LRT were not identified by the global model, and vice versa. Examination of the expression data revealed that the global model was often able to identify differentially expressed spots when the probability of expression was low in both groups. This is most likely due to the fact that the LRT does not have sufficient power to detect differences when sample sizes in both groups are small. In contrast, the global model uses a common variance (obtained across all spots) for expression intensities, and this allows inferences to be made even when sample sizes are low in both groups.

Of course, because the global model uses a common variance for expression intensities, spots where the variances are significantly different from the common variance will not necessarily be identified as differentially expressed. This appears to account for those spots that are identified by the LRT and not the global Bayesian analysis.

## Conclusions

We have demonstrated with simulated data that a global Bayesian model is able to correctly identify more differentially expressed proteins than the use of the LRT proposed in the previous study. In all three simulation analyses, the LRT classified approximately 60% of the proteins as statistically differentially expressed, and the global model classified between 75% and 89% of the proteins. Additionally, with our simulated data, the global model identified correctly identified almost all of the proteins also identified by the LRT. The global model accurately recovered the underlying global distributions in all simulations. The 95% HPD for the five global parameters, *μ*_*g*_, *σ*_*g*_, *ψ*, *λ*_*δ*_ and *ϕ*_*δ*_, always included the true values used to simulate the dataset. The global distributions used in the model were fixed, but the results from the simulation analyses showed that it can be adapted to a wide range of different underlying distributions. In simulation analysis 2, the model recovered a wide normal distribution to overcome the fact that the underlying distribution was a uniform distribution. In simulation analysis 3, a very wide normal distribution with standard deviation 3.65 was obtained when two non-overlapping normal distributions were used as the true distributions from which data were sampled. Finally, simulations also demonstrated that the False Discovery Rate was very low.

When we applied the global Bayesian analysis and the LRT to real data, we uncovered some interesting disparities that appear to be related to how these methods apply variance estimates. In particular, the global Bayesian model estimates a common variance by combining data available from all spots. This allows the model to estimate the standard deviation more accurately if there is, indeed, a common variance of expression intensities. By using the 95% HPD to identify differentially expressed proteins, additional information is provided on whether a protein is differentially expressed due to the expression intensities, probabilities of expression or possibly both. The proportion of up- or down-regulated proteins can be accurately estimated from the model by the global parameter *ϕ*_*δ*_. In contrast, the LRT uses only the variance of expression intensities identified for each spot. If the number of expressed spots is low in both Case and Control groups, the power to detect differences is compromised. This is an advantage of the global model when the assumption of a common variance is appropriate. However, when this assumption is violated, the global model does not identify the same spots as being up- or down-regulated as the LRT. It may be possible to apply a mixture of distributions allowing different variances, to overcome this discrepancy. However, it is a common to find with MCMC procedures that adding more parameters, and integrating over these, affects mixing and convergence to the stationary distribution.

It is, of course, true that a realistic biological system involves several different groups of proteins, with each group associated with different biological pathways that are frequently interconnected. In order to capture this complex relationship, it is likely that the expressions of different clusters of proteins will be best explained by different underlying distributions. This will allow the model to separate proteins into several different categories, with each category being represented by a unique global distribution. Whereas the use of multiple global distributions may result in a more accurate estimate of these true global parameters, there is also the danger that introducing new distributions (and new parameters) will lead to overfitting and inflated variance estimates. Several global statistical models developed for other high throughput technologies such as microarrays, often attempt to incorporate biological pathways [22]. The challenge with 2D PAGE is that the true identity of each protein is usually unknown until differentially expressed proteins are determined and then subjected to mass spectroscopy for identification. Without this information, it is very challenging to develop a global model based on biological pathways.

Finally, one other assumption that our global Bayesian model makes is that the variances of expression intensities for the Case and Control groups are equal. We are aware that this may be an unrealistic assumption; however, if we assume the alternative (i.e., unequal variances for Case and Control), our implementation of the MCMC has difficulty converging when the probability of expression is low.

Any MCMC Bayesian analysis requires a choice of prior distributions. Although we have designed priors that appear to be a reasonable characterization of the uncertainty in our parameter values, the model is general enough to allow other priors to be substituted for the ones we use. In this paper, we have not tried different prior distributions, because we are demonstrating how the Bayesian MCMC scheme may be implemented, and we have applied our methods largely to simulated data. With real-world data, it is standard practice when applying Bayesian analyses to real data to test for the sensitivity to different prior distributions.

One drawback of the MCMC approach is the amount of time required for the Markov chain to converge. Multiple runs of Markov chains can be used to assess the convergence and accuracy of the results. An example of this is the Metropolis-coupled Markov chain Monte Carlo (MC^3^) approach [23]. A typical 2D PAGE experiments may have between 800 to 1200 expressed proteins. With the current implementation, it took around 1.7 hours per million iterations for an experiment with 800 spots on a Intel i5 2.67 GHz CPU. As the number of spots increases, the number of iterations and the time required for the Markov chain to converge may also increase. To improve the usability of this model, a more efficient implementation, such as parallel MCMC, should be used [24]. The source code and jar file are available for download at https://github.com/stevenhwu/BIDE-2D.

## Competing interests

The authors declare that they have no competing interests.

## Authors' contributions

SHW, MAB and AGR conceived and designed the model. SHW performed the analysis. RAN Contributed the data. SHW, MAB, RAN and AGR wrote the manuscript. All authors read and approved the final manuscript.
